# Malonate as a ROS product is associated with pyruvate carboxylase activity in acute myeloid leukaemia cells

**DOI:** 10.1186/s40170-016-0155-7

**Published:** 2016-08-04

**Authors:** Michelle A. C. Reed, Christian Ludwig, Christopher M. Bunce, Farhat L. Khanim, Ulrich L. Günther

**Affiliations:** 1 Institute of Cancer and Genomic Sciences, University of Birmingham, Birmingham, B15 2TT UK; 2 School of Biosciences, University of Birmingham, Birmingham, B15 2TT UK; 3 Institute of Metabolism and Systems Research, University of Birmingham, Birmingham, B15 2TT UK

**Keywords:** Cancer metabolism, Pyruvate carboxylase, AML, Malonate

## Abstract

**Background:**

The role of anaplerotic nutrient entry into the Krebs cycle via pyruvate carboxylase has been the subject of increased scrutiny and in particular whether this is dysregulated in cancer. Here, we use a tracer-based NMR analysis involving high-resolution ^1^H-^13^C-HSQC spectra to assess site-specific label incorporation into a range of metabolite pools, including malate, aspartate and glutamate in the acute myeloid leukaemia cell line K562. We also determine how this is affected following treatment with the redeployed drug combination of the lipid-regulating drug bezafibrate and medroxyprogesterone (BaP).

**Results:**

Using the tracer-based approach, we assessed the contribution of pyruvate carboxylase (PC) vs. pyruvate dehydrogenase (PDH) activity in the derivation of Krebs cycle intermediates. Our data show that PC activity is indeed high in K562 cells. We also demonstrate a branched entry to the Krebs cycle of K562 cells with one branch running counterclockwise using PC-derived oxaloacetate and the other clockwise from the PDH activity. Finally, we show that the PC activity of K562 cells exclusively fuels the ROS-induced decarboxylation of oxaloacetate to malonate in response to BaP treatment; resulting in further Krebs cycle disruption via depletion of oxaloacetate and malonate-mediated inhibition of succinate dehydrogenase (SDH) resulting in a twofold reduction of fumarate.

**Conclusions:**

This study extends the interest in the PC activity in solid cancers to include leukaemias and further demonstrates the value of tracer-based NMR approaches in generating a more accurate picture of the flow of carbons and metabolites within the increasingly inappropriately named Krebs cycle. Moreover, our studies indicate that the PC activity in cancer cells can be exploited as an Achilles heel by using treatments, such as BaP, that elevate ROS production.

**Electronic supplementary material:**

The online version of this article (doi:10.1186/s40170-016-0155-7) contains supplementary material, which is available to authorized users.

## Background

We have previously demonstrated the individual and combined anti-proliferative and pro-differentiating actions of a drug combination termed bezafibrate and medroxyprogesterone (BaP), consisting of the lipid-regulating drug bezafibrate (BEZ) and the steroid contraceptive medroxyprogesterone (MPA) against primary acute myeloid leukaemia (AML) cells and cell lines [1–4], Burkitt’s lymphoma (BL) cell lines [5] and primary chronic lymphocytic leukaemia cells [6]. In addition, phase II trials of BaP in both AML and BL have demonstrated in vivo anti-tumour activity in the absence of toxicity [7, 8] and, in the case of AML, generated significant haematological responses in some patients [7].

We previously demonstrated that BaP treatment of KG1α, K562 and HL60 AML cell lines was associated with excess generation of reactive oxygen species (ROS), inducing a range of metabolic changes involving pathways related to the Krebs cycle, specifically increased succinate/fumarate ratios. The importance of ROS for novel therapies has also been recognised by others [9–11] who linked ROS to iron homeostasis [10] and AKT phosphorylation [11]. Our observations also implicated direct ROS-mediated chemical conversion of metabolites including the conversion of α-ketoglutarate (α-KG) into succinate and of oxaloacetate to malonate [4, 12, 13]. Malonate is known to inhibit succinate dehydrogenase [14], thus interrupting the conversion of succinate to fumarate.

Here, we present a detailed tracer-based analysis of metabolism in K562 AML cells with and without exposure to BaP to decipher the origin of malonate and the relative contributions of pyruvate carboxylase (PC) and pyruvate dehydrogenase (PDH) activity for the entry of nutrients into the Krebs cycle. In order to determine site-specific label incorporations, we used NMR ^1^H-^13^C-HSQC spectra. In such spectra, every signal arises from a CH group in a metabolite. When acquired with sufficiently high resolution in the ^13^C-dimension, one can resolve couplings arising from adjacent ^13^C atoms providing crucial information about site-specific label incorporation. Previously, we have used this approach to study the distribution of ^13^C-labels arising from [1,2-^13^C]glucose and from [3-^13^C]glutamine in K562 cells [15]. Here, we have used this analysis to trace the origin of malonate. Our analysis also sheds new light on pyruvate carboxylase activity in cancer cells, an issue that has been raised in various tumours [16–18].

## Methods

### Sample preparation

 K562 cells were cultured and polar cell extracts were prepared as described before [4]. For tracer-based metabolic analysis, standard glucose- or glutamine-free RPMI-1640 media (Gibco) was used and substituted with 2 g/l [1,2-^13^C]glucose or 300 mg/l [3-^13^C]glutamine (Isotec, Sigma), respectively. 5 × 10^7^ exponentially growing K562 cells per control or treatment were pelleted by centrifugation at 8000*g* for 5 min and resuspended in the relevant media with BaP or solvent controls and incubated for 24 or 3 h at 37 °C and 5 % CO_2_ in a humidified incubator. For BaP treatment, bezafibrate 0.5 mM and medroxyprogesterone acetate 5 μM (Sigma) or the equivalent concentrations of DMSO and ethanol solvent control were added to the media at *T* = 0 h.

For 3-h labelling of 24-h drug treatments, media was exchanged for the last 3 h with media supplemented with labelled glucose or glutamine plus BaP or solvent control. All samples were dissolved in 100 mM phosphate buffer with 10 % D_2_O and 500 μM Trimethylsilylpropanoic acid (TMSP) added.

### NMR data acquisition

All spectra were acquired at 298 K on a Bruker 600 MHz spectrometer with a TCI 1.7 mm z-PFG cryogenic probe. ^1^H-^13^C-HSQC spectra were acquired using the Bruker sequence hsqcetgpsp.2 with added gradients during echoes, using 4096 points in the directly observed dimension for a sweep width of 13 ppm. Four thousand ninety-six increments, two scans and an inter-scan delay of 1.5 s were used. The ^13^C carrier was set to 80 ppm and a spectral width 159.0 ppm was used.

### Processing of NMR spectra

All one-dimension spectra were processed using NMRLab [19] in MATLAB and further analysed using MetaboLab [20] as described before [4]. In HSQC spectra peaks were picked and assigned in a semi-automated manner using MetaboLab. To calculate percentage label incorporations, the cross peaks in labelled spectra and reference spectra were compared. The ^13^C isotope constitutes about 1 % of naturally occurring carbon. Therefore, for more concentrated metabolites, cross peaks could be seen in the reference spectra. Peak intensities in control and reference spectra were used to calculate percentage incorporation of labels into particular carbons of metabolites as follows:

The percentage incorporation of ^13^C into peak *X* of metabolite *Y* in ^13^C-labelled media % equals 100 N/(D*S), where % = percentage incorporation, *N* = intensity of a signal of a metabolite in labelled media spectrum, *D* = intensity of a signal of a metabolite in the control spectrum, *S* = mean(ScaleFactor). ScaleFactor(*i*) = Nr/Dr, where Nr = intensity of peak *i* from reference metabolite in numerator spectrum, Dr = intensity of peak *i* from reference metabolite in denominator spectrum.

A reference metabolite was chosen as one of a group of metabolites that did not change intensity significantly between BaP and control spectra or between spectra for enriched and natural abundance media. Results were similar using myo-inositol, valine, leucine or isoleucine as the reference metabolite. Labelling was not considered significantly changed unless BaP treatment changed percentage label incorporation by a factor of 2.

For signals in spectra arising from [1,2-^13^C]glucose-labelled cells that showed CC-couplings, the peak intensity was the sum of the intensities for the split peaks. In some spectra, peaks could not be seen. In such cases, the peak intensity was set to the estimated noise level in that spectrum. That noise level is evaluated by searching for the maximum of the absolute value of the intensity seen in a region devoid of real signals.

When calculating percentage incorporations of ^13^C, it is the natural abundance reference spectrum peak intensity that may be missing. Substituting the dummy peak intensity tends to cause the percentage incorporation of ^13^C to be (conservatively) underestimated.

### Malonate spiking

For spiking, a sample from a BaP-treated HL60 or K562 cell extract grown on unlabelled media was split into two. To the first sample, metabolomics buffer was added. To the second sample, an equal amount of metabolomics buffer containing 1 mM malonic acid was added. ^1^H-1D and ^1^H-^13^C-HSQC spectra were acquired. Spectra were aligned on TMSP and lactate, respectively, for overlay in 1D and 2D spectra.

## Results

**Fig. 1:**
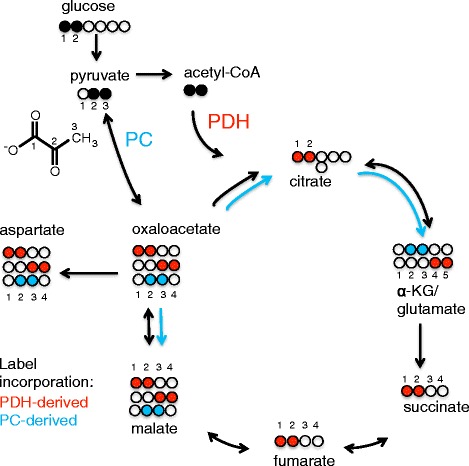
Label distribution arising from [1,2-^13^C]glucose via pyruvate dehydrogenase (PDH) and pyruvate carboxylase (PC), respectively. For PDH (*red*), label incorporation in a clockwise direction is assumed. For PC (*blue*) label incorporation is considered in an anti-clockwise direction, except for clockwise labelling of glutamate. For the onward processing from α-ketoglutarate in a clockwise direction, the loss of C1 produces [1,2-^13^C]succinate which is, owing to the symmetry of succinate, identical to the PDH-derived product

We employed [1-^13^C]glucose, [1,2-^13^C]glucose and [3-^13^C]glutamine nutrient sources to decipher the carbon flow in K562 cells and changes in response to BaP treatment. Overall, we observe the largest amount of label incorporation into ribose moieties and lactate (see [15] and Additional file 1: Figure S1 for details on label distributions). As illustrated in Fig. 1, label incorporation into the Krebs cycle arising from [1,2-^13^C]glucose can be expected to produce two distinctly different labelling patterns of Krebs cycle metabolites depending upon whether the C2 fragment enters via pyruvate carboxylase (PC) or pyruvate dehydrogenase (PDH). The [2,3-^13^C]pyruvate formed from [1,2-^13^C]glucose will yield [4,5-^13^C]glutamate via PDH, but [2,3-^13^C]glutamate via PC activity. As PC activity produces oxaloacetate from pyruvate, one expects [2,3-^13^C]malate derived from PC, whereas the PDH product will be [1,2-^13^C]malate or [3,4-^13^C]malate.

### NMR spectra indicate that large amounts of aspartate and malate are PC derived

**Fig. 2:**
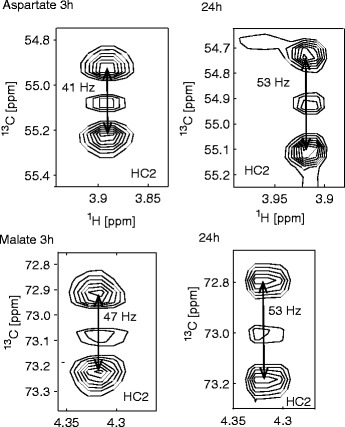
Sections from HSQC spectra for K562 cells labelled with [1,2-^13^C]glucose showing peak splittings arising from the *J*_CC_ coupling. Spectra are shown for the HC_2_ atoms of aspartate and malate at 3 and 24 h labelling, showing different sizes of apparent coupling constants

As shown in Fig. 2, significant differences are observed between cells processing [1,2-^13^C]glucose for 3 vs. 24 h for the resonances of malate and aspartate. These differences manifest predominantly in a change of NMR-coupling constants. The size of these coupling constant depends on the nature of the adjacent carbon atom: a carboxylic acid group yields a much larger coupling constant than a CH_2_ group. The coupling constant for the ^13^C_1_^13^C_2_ moiety in aspartate or malate is about 50–60 Hz whereas the coupling constant for a ^13^C_2_^13^C_3_ moiety is about 35–40 Hz.

For a 24-h labelling period, Fig. 2 shows apparent coupling constants of 53 Hz for C_2_ of malate and aspartate. This large apparent coupling constant shows that the coupling of C_2_ with the adjacent C_1_ carboxylic acid group predominates. This ^13^C_1_^13^C_2_ coupling pattern (and also ^13^C_3_^13^C_4_, see Additional file 1) arises from the PDH activity and possibly also from metabolites passing multiple times through the Krebs cycle for longer labelling period.

For the short 3-h labelling period, the smaller apparent coupling constants of 47 and 41 Hz are observed for C_2_ (Fig. 2 and Additional file 1: Figure S1), indicating that the coupling to the adjacent methylene C_3_ dominates. The presence of the ^13^C_2_^13^C_3_ moiety at shorter labelling periods shows that the product arising from the PC activity dominates for shorter labelling periods.

It should be noted that the observed signal splittings do not represent the precise scalar coupling constants in a mixture of labelled compounds. Nevertheless, the observed change provides a clear indication of higher amounts of the PC product at shorter labelling periods in both malate and aspartate.

### Further evidence for PC activity in subspectra of uracil

In de novo pyrimidine synthesis, uracil is formed from aspartate via carbamoyl-aspartate, orotate and dihydroorotate. Therefore, labelling patterns in aspartate should be reflected in pyrimidine ring signals. Additional file 2: Figure S2 shows expected label incorporations for uracil. When the uracil base in UDP is synthesised from aspartate, the destination of the ^13^Cs is as follows: C_1_, C_2_ and C_3_ of aspartate become respectively C_10_, C_11_ and C_12_ of UDP while C_4_ is lost (see Additional file 2). In HSQC spectra, only C_11_ and C_12_ are able to be directly observed, as C_10_ does not have an attached proton.

For [1,2-^13^C]glucose-labelled samples the PC-derived aspartate will be C_2_C_3_-labelled. This is converted to a labelled C_11_C_12_ fragment in UDP. However, PDH-derived aspartate can be labelled at C_1_C_2_ or C_3_C_4_. This translates into C_10_C_11_ or an isolated C_12_ in uracil, respectively. Therefore, spectra of C_12_ are indicative of relative amounts of PC vs. PDH activity; PC yields a doublet for C_12_, whereas PDH yields a singlet at C_12_.

**Fig. 3:**
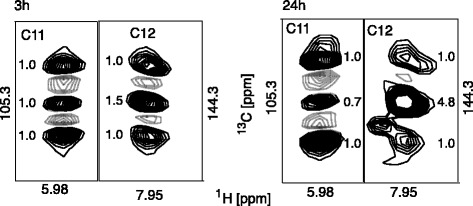
Signals observed in [1,2-^13^C]glucose-labelled cells for UDP, showing different intensities (numbers besides signals) for 3 and 24 h labelled cells

All spectra showed both the singlet and doublet signals at C_12_ (Fig. 3). However, the intensity of the doublet relative to the singlet changed between 3 and 24 h data by a factor of three, again indicating a shift from PC to PDH-mediated labelling with time. The picture that emerges from several metabolites is that there is parallel activity of both PC and PDH, but the PC product is primarily channelled into products directly linked to oxaloacetate, yielding the high observed amount of PC product into these metabolites in a short-term exposure. Only at longer labelling periods is the PDH product observed in the left branch of the Krebs cycle.

### BaP-induced malonate is derived from downstream PC activity in K562 cells

We have shown that malonate can be formed from oxaloacetate by chemical conversion under the influence of hydrogen peroxide and suggested in our previous study that malonate accumulation in response to BaP treatment was driven by treatment-induced elevation of ROS acting upon oxaloacetate [4]. Samples were spiked with malonate to confirm our assignment of the malonate methylene ^1^H/^13^C resonances in HSQC spectra (see Additional files 3 and 4). Subsequently, in this study, our tracer-based approach has allowed us to consider the origins of the observed malonate.

**Fig. 4:**
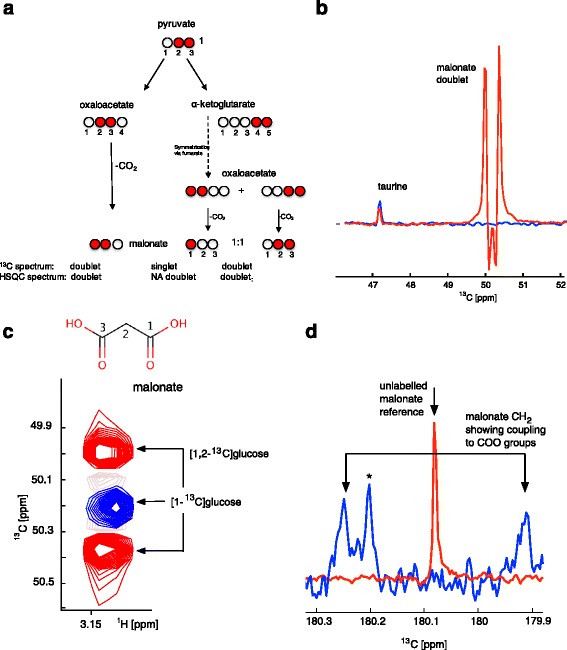
**a** Expected labelling patterns in malonate derived from oxaloacetate by decarboxylation and expected signal patterns in directly observed ^13^C spectra. **b** Slices from HSQC spectra for malonate for [1,2-^13^C]glucose-derived samples with (*red*) and without (*blue*) BaP treatment. **c** Peak patterns observed for malonate in ^1^H-^13^C-HSQC spectra, *red* for [1,2-^13^C]glucose-labelled cells, *blue* for [1-^13^C]glucose-labelled cells. **d** ^13^C-NMR spectra for the carboxylic acid region showing the spectrum arising from [1,2-^13^C]glucose with BaP in *blue* and the reference spectrum of unlabelled malonate in *red*. The lack of a centre peak proves that ^13^COO is always adjacent to a labelled CH_2_. The *asterisk* denotes a non-malonate-derived carbon atom

As shown in Fig. 4a, ROS-derived malonate arising from oxaloacetate that had been formed directly from pyruvate by PC activity using [1,2-^13^C]glucose as the nutrient source would be expected to be labelled in positions C_1_ and C_2_. In the alternative situation that PDH activity converts pyruvate into acetyl-coA upon entry into the Krebs cycle, ROS-mediated conversion of the resulting oxaloacetate into malonate would be expected to give rise to two products with label in the C_1_ or in the C_1_ and C_2_ position in a 1:1 ratio because the Krebs cycle passes through symmetrical succinate and fumarate (see also Fig. 1).

Figure 4b shows a representative 1D slice from HSQC spectra arising from 24 h BaP-treated [1,2-^13^C]glucose-labelled cells and clearly demonstrates drug-induced generation of a strong malonate signal. In the corresponding HSQC spectra arising from 24 h BaP-treated [1,2-^13^C]glucose-labelled cells, we observed a clear doublet (Fig. 4c shown as red peaks) with a splitting of 58 Hz, indicative of a labelled CH_2_ group coupled to a carboxylic acid carbon. This is a clear indication of a C_1_,C_2_ (or C_2_,C_3_)-labelled malonate with label in only one of the two carboxylic acid groups. Malonate labelled in all three positions that might arise from multiple passages through the Krebs cycle would be expected to show a triplet at the C_2_ in the ^13^C-dimension of HSQC spectra, as at least some percentage would be labelled in both COO groups (AX_2_ coupling pattern). Therefore, the absence of a central signal in HSQC is highly indicative that malonate is derived from upstream PC activity. The absence of malonate derived from multiple Krebs cycles and PDH activity is also supported by the fact that malonate arising from 24 h labelling of BaP-treated cells with [1-^13^C]glucose showed only a singlet (Fig. 4c shown as blue peak).

In order to further prove that drug-induced malonate is derived downstream of PC activity, we acquired ^13^C-1D-spectra in which we could observe the COO resonances of malonate directly (Fig. 4d). A reference spectrum of 10 mM malonic acid in the same (pH 7) buffer as used for cell extracts confirmed the frequency of the COO resonance (Fig. 4d).

PC-mediated labelling is expected to yield a doublet at C_1_ (arising from [1,2-^13^C]malonate), whereas PDH-mediated labelling is expected to give a 50:50 mixture of a doublet arising from [1,2-^13^C]malonate and a singlet arising from [1-^13^C]malonate (Fig. 4a). Although noisy even after 24 h of acquisition, the spectra derived showed a clear doublet with no residual signal in the middle (Fig. 4d) confirming that the PC-derived labelling product is dominant. From this, we conclude that malonate is indeed derived from ROS-mediated conversion of oxaloacetate originating entirely or at least predominantly from the PC activity and does not show any contribution from a possible PDH product even after a 24 h labelling period. Control experiments using [3-^13^C]glutamine as a carbon source showed only minimal label incorporation into malonate. The malonate produced from [1,2-^13^C]glucose was shown to be predominantly labelled via PC. Together, these two facts strongly suggest that malonate is produced predominantly from glucose via glycolysis and PC-mediated entry of pyruvate into the TCA cycle.

### Evidence of parallel PDH activity

Table 1 compares ^1^*J*_CC_ coupling constants and multiplet intensity patterns seen in the 3 and 24 h labelled datasets. In both the 3 and 24 h data sets, labelling at glutamate’s C_4_ is much greater than the labelling at C_4_ and the splitting seen at C_4_ indicates coupling to C_5_. This strongly suggests that PDH-mediated labelling is dominant for glutamate at both time points. The citrate C_2_ is split by a large coupling to C_1_, again strongly suggesting that PDH-mediated labelling is dominant. These labelling patterns indicate a clear dominance of PDH products for the right branch of the Krebs cycle, leading from citrate to glutamate.

**Table 1:** Observed coupling constants in ^1^H-^13^C-HSQC spectra

	3 h		24 h	
Metabolite and atom at which coupling is observed	Control	BaP	Control	BaP
Aspartate	41:47:41	47:41:41	53:53:53	53:53:53
C2:C3a:C3b ^1^ *J*_CC_/Hz				
Malate	47:47:41	47:47:47	53:53:53	59:53:59
C2:C3a:C3b ^1^ *J*_CC_/Hz				
Fumarate C2	Singlet	Singlet	70	70
^1^ *J*_CC_/Hz				
Succinate C2	53	59	59	59
^1^ *J*_CC_/Hz				
Glutamate	53:41:59	53:41:59	59:35:59	59:35:53
C2:C3:C4 ^1^ *J*_CC_/Hz				
Citrate C2a:C2b	59:53	65:65	59	59
^1^ *J*_CC_/Hz				

### Computational multiplet analysis

Slices from the ^1^H-^13^C-HSQC were also quantitatively analysed by simulating ^13^C-NMR spectra for a mixture of different isotopomers using the pyGamma software [21] from within the NMRLab software [19]. For glutamate, the multiplet analysis confirmed that PDH-mediated labelling was dominant at both 3 and 24 h irrespective of BaP treatment. For aspartate, the multiplet analysis confirmed that there was a shift from PC-mediated labelling at 3 h towards PDH-mediated labelling at 24 h. The simulated spectra are shown in Additional file 5: Figure S4, and Table 2 confirms qualitative results for aspartate and glutamate.

**Table 2:** Percentages of isotopomers arising from a computational multiplet analysis

3 h Labelling				
	Control		BaP treated	
Asp	%PDH	%PC	%PDH	%PC
	1.28 ± 0.08	2.11 ± 0.04	1.52 ± 0.17	2.25 ± 0.11
Glu	%PDH	%PC	%PDH	%PC
	2.99 ± 0.04	0.64 ± 0.04	4.63 ± 0.07	0.68 ± 0.09
24 h labelling				
	Control		BaP treated	
Asp	%PDH	%PC	%PDH	%PC
	3.17 ± 0.31	0.86 ± 0.08	2.62 ± 0.15	1.03 ± 0.17
Glu	%PDH	%PC	%PDH	%PC
	4.42 ± 0.14	0.66 ± 0.08	5.32 ± 0.29	0.58 ± 0.06

## Discussion

This study sheds further light on the action of ROS in AML cell lines. The importance of ROS for the treatment of leukaemic cancers has previously been highlighted by us [4, 22] and by others [9–11]. Our previous study also showed that high levels of ROS are associated with chemical conversion of oxaloacetate into malonate, and this phenomenon is common to a variety of AML cell lines [4].

A number of studies, including some very recent reports, have investigated PC activity in relation to cancer. DeBerardinis and coworkers showed that PC takes over as the alternative anaplerotic mechanism when glutaminolysis is silenced [23]. This is seen in the labelling pattern obtained for glutamate C_2_ in tracer based metabolic analyses using glioblastoma cell lines. PC has been shown to be enhanced in human non-small cell lung cancers in xenograft mouse models [24]. It has also been shown that PC activity is high in human lung tumours [16, 17, 25] and critical for cell proliferation and colony formation in human non-small cell lung cancer cells [18]. Likewise, Phannasil et al. showed that PC was upregulated in breast cancer tissues, PC expression was higher in cell lines with greater metastatic potential, and that proliferation, migration and in vitro invasion ability is PC dependent [26]. Recent work shows that pyruvate carboxylation diverts glucose-derived carbons into aspartate biosynthesis in succinate dehydrogenase (SDH)-ablated kidney mouse cells [27]. In analogy to non-small cell lung cancers, we also observe high PC activity in the AML K562 cell line.

The patterns of metabolite labelling we observed and the kinetics of their changes with time lead us to conclude that the Krebs cycle is disrupted in K562 cells. We have clearly demonstrated the branched uptake of pyruvate into the cycle via ‘left-hand’ PC-mediated and ‘right-hand’ PDH-mediated entry. The PC-mediated entry was readily observed at smaller labelling periods (3 h). At this time, the conversion of PDH products in the right-hand branch of the Krebs cycle into malate and aspartate was low but became more evident at 24 h.

However, continued PC-mediated entry to the Krebs cycle even at longer flux periods was confirmed by the origin of malonate following BaP treatment. Our earlier study identified malonate accumulation in response to BaP. However, [1-^13^C]glucose and [1,2-^13^C]glucose trace labelling approaches have demonstrated that this malonate unexpectedly originates almost exclusively from PC-derived oxaloacetate.

The generation of malonate in response to BaP treatment, in turn appears to drain the pool of oxaloacetate being formed from PC activity. At the same time, the accumulated malonate, which is known to block the SDH activity, appears to further disrupt the Krebs cycle as evidenced by a twofold reduction in the fumarate/succinate ratio following BaP treatment.

Importantly, we did not observe any visible contribution to the malonate pool from any PDH-derived products after one or several complete passages through the Krebs cycle providing remarkable evidence of direct conversion of oxaloacetate to malonate at the site of its formation by PC.

Whether the mechanisms described here require specific niche conditions, such as those in peripheral blood, cannot be answered from the existing study. Preclinical studies of BaP anti-cancer activities were demonstrated in non-hypoxic cultures [8] and translated to clinical efficacy in vivo including haematological responses as well as reduction in tumour load [7, 8]. As malonate formation under high ROS has been observed in several AML cell lines and can be reproduced by treatment of cell extracts in vitro [4], we suggest that the role of malonate may be common to any cell when ROS is sufficiently high.

## Conclusions

In a wider context, this study indicates that in the case of AML cells, malonate represents a marker of increased ROS, an observation that needs investigation in other cancer models. This is also important for the wider application of our findings for cancer treatments. As discussed above, there are a growing number of observations indicating that PC activity underpins the neoplastic characteristics of several cancers. Using BaP here as an example, we have shown that the PC activity of cancer cells can be exploited, as an Achilles heel, by fuelling ROS-generated production of malonate. On a broader perspective in cancer, this in turn has implications for the development of malonate derivatives as potential cancer therapeutics.

## Abbreviations

AML, acute myeloid leukaemia; BaP, bezafibrate and medroxyprogesterone; BL, Burkitt’s lymphoma; HSQC, heteronuclear single quantum coherence; PC, pyruvate carboxylase; PDH, pyruvate dehydrogenase; ROS, reactive oxygen species; SDH, succinate dehydrogenase

## Additional files

Additional file 1: Figure S1. Peak patterns observed for aspartate HC_2_ and HC_3_. Sections from HSQC spectra for K562 cells labelled with [1,2-^13^C]glucose showing peak splittings arising from the *J*_CC_ coupling. Spectra are shown for the HC_2_ and HC_3_ atoms of aspartate at 3 and 24 h labelling, showing different sizes of apparent coupling constants. (PDF 1.03 mb) Additional file 2: Figure S2. Label incorporation into pyrimidines. Label distribution arising from [1,2-^13^C]glucose via pyruvate dehydrogenase (PDH) in red; from [1,2-^13^C]glucose via pyruvate carboxylase (PC) in purple; and from [3-^13^C]glutamine in green, respectively. Note that the PDH route produces an isolated ^13^C at C_12_ in UDP. (PDF 1.03 mb) Additional file 3: Figure S3A. Malonate spiked sample. An unlabelled cell extract was split into two, and to one sample, buffer containing malonate acid was added, and to the other sample, an equal volume of buffer was added. Regions from the resulting ^1^H NMR spectra for the original and spiked samples are overlaid in blue and red, respectively. (PDF 1.03 mb) Additional file 4: Figure S3B. Malonate spiked sample. An unlabelled cell extract was split into two, and to one sample, buffer containing malonic acid was added and the pH adjusted to 7, and to the other sample, an equal volume of buffer was added. The regions of the resulting HSQC spectra containing the malonate and lactate resonances for the original and spiked samples are overlaid in blue and red, respectively. Lactate is shown as a sensitivity reference. (PDF 1.03 mb) Additional file 5: Figure S4. Simulation of spectral multiplets arising from ^1^H-^13^C-HSQC spectra for aspartate and glutamate. (PDF 383 kb)
