# Supplementary material for: Malonate as a ROS product is associated with pyruvate carboxylase activity in acute myeloid leukaemia cells
Source: Cancer Metab. 2016 Aug 4;4:15. doi: 10.1186/s40170-016-0155-7 (PMC4972992; doi:10.1186/s40170-016-0155-7)

Supplementary Material

Relative peak intensities in UDP

Overall the largest fraction of label arising from [1,2-^{13}C]glucose is observed in riboses and lactate. A detailed list of label incorporation values is given in [1]. As lactate (and other metabolites) can be secreted into the media label incorporation values from cell extracts don't reflect the overall flux of label.

In order to assess the relative distribution of label between PPP and the Krebs cycle we have looked at label incorporation in pyrimidine nucleotides which contain a ribose moiety labelled via PPP activity and an aspartate-derived pyrimidine ring moiety labelled via glycolysis and the Krebs cycle. The summed peak intensities for different carbons in UDP species are as follow:

C1 164
C2 57
C4 40
C5 46
C11 24
C12 26

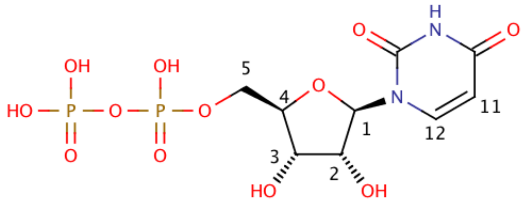

Peak intensities for lactate and alanine:

Lactate C2 294; C3 (doublet) 1416
Alanine C2 2; C3 13

Summed peak intensity in ribose carbons of UDPs is 307; summed peak intensity in aspartate-derived pyrimidine carbons is 50. This shows that the largest amount of label flows into lactate and PPP derived riboses.

1. Carrigan JB, Reed MAC, Ludwig C, Khanim FL, Bunce CM, Günther UL. Tracer-Based Metabolic NMR-Based Flux Analysis in a Leukaemia Cell Line. ChemPlusChem. 2016.

Figure S1: Peak patterns observed for aspartate HC2 and HC3

Aspartate 3h

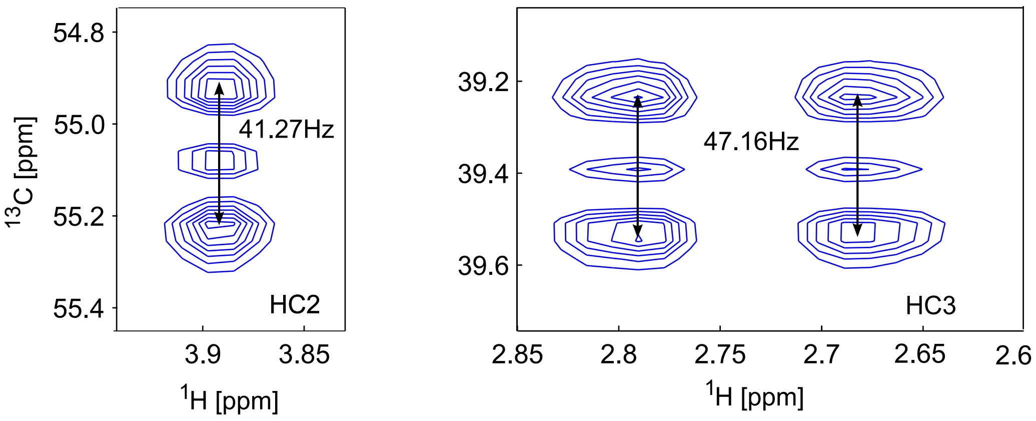

Aspartate 24h

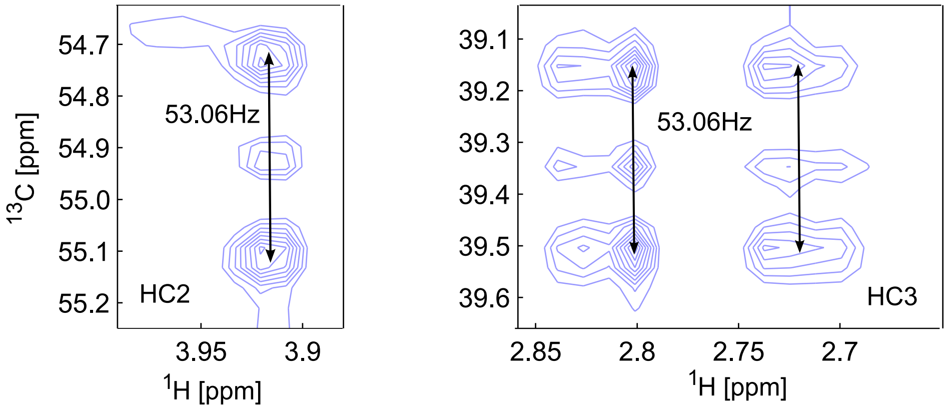

Aspartic acid,

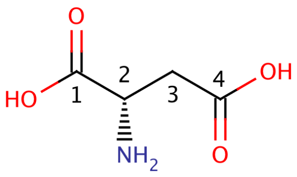

Supplement: Additional file 1: Figure S1. — Peak patterns observed for aspartate HC2 and HC3. Sections from HSQC spectra for K562 cells labelled with [1,2-13C]glucose showing peak splittings arising from the J CC coupling. Spectra are shown for the HC2 and HC3 atoms of aspartate at 3 and 24 h labelling, showing different sizes of apparent coupling constants. (PDF 1.03 mb) [file 40170_2016_155_MOESM1_ESM.pdf]
