# Supplementary figures and images for: Malonate as a ROS product is associated with pyruvate carboxylase activity in acute myeloid leukaemia cells
Source: Cancer Metab. 2016 Aug 4;4:15. doi: 10.1186/s40170-016-0155-7 (PMC4972992; doi:10.1186/s40170-016-0155-7)

**Figure S2: Label Incorporation into pyrimidines**

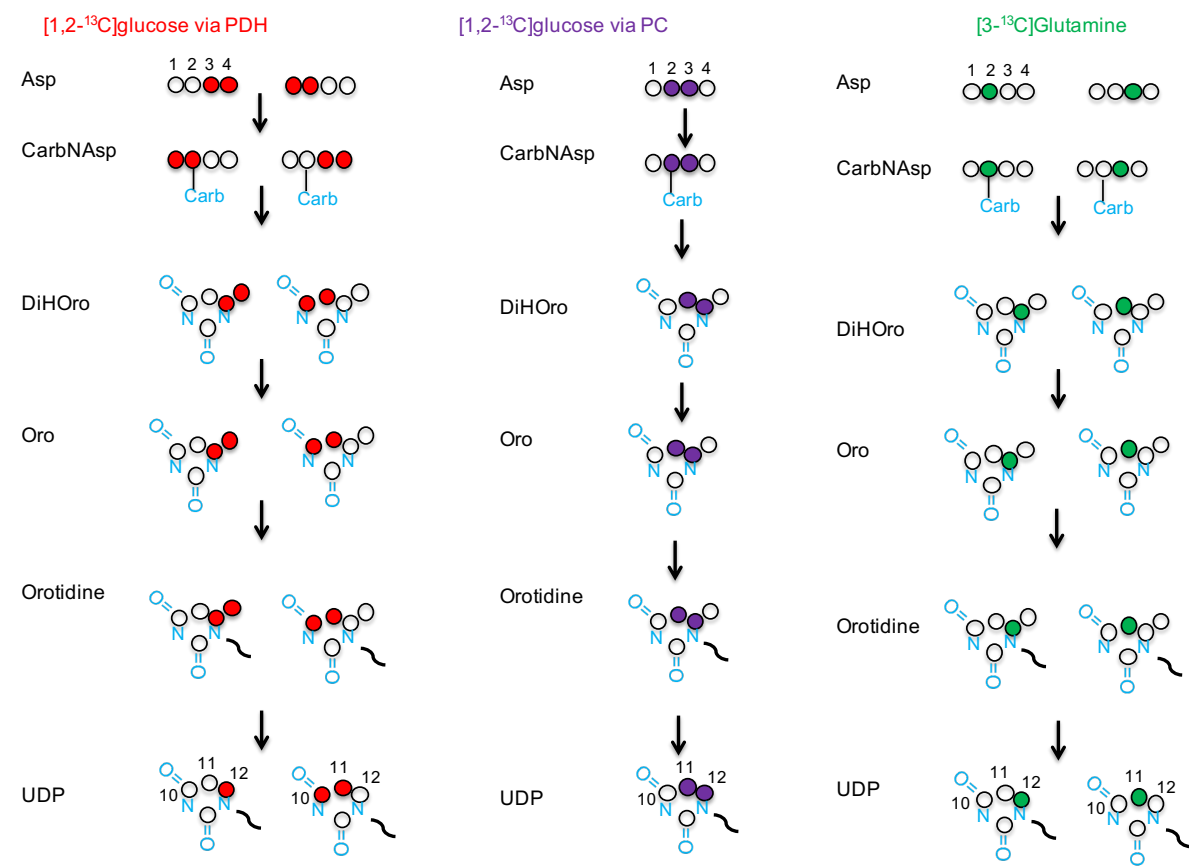

Supplement: Additional file 2: Figure S2. — Label incorporation into pyrimidines. Label distribution arising from [1,2-13C]glucose via pyruvate dehydrogenase (PDH) in red; from [1,2-13C]glucose via pyruvate carboxylase (PC) in purple; and from [3-13C]glutamine in green, respectively. Note that the PDH route produces an isolated 13C at C12 in UDP. (PDF 1.03 mb) [file 40170_2016_155_MOESM2_ESM.pdf]

**Figure S3A: 1D  $^1\text{H}$ -NMR spectrum of a malonate spiked sample**

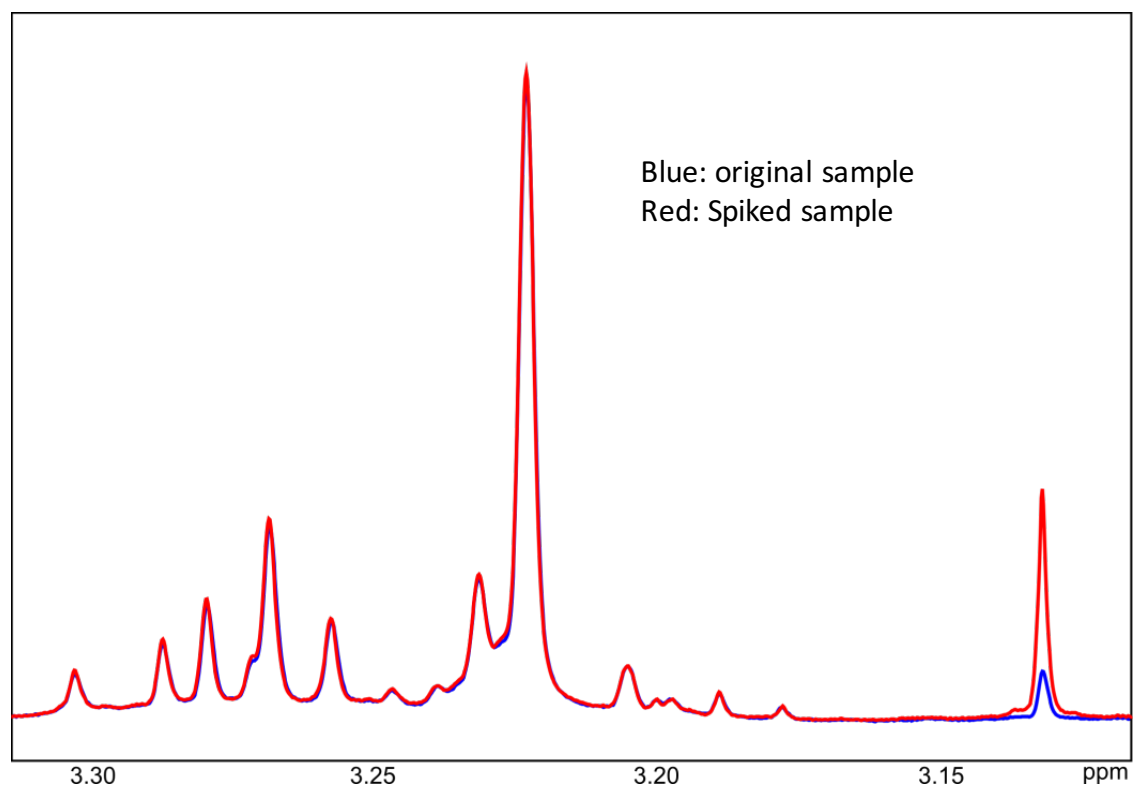

Supplement: Additional file 3: Figure S3A. — Malonate spiked sample. An unlabelled cell extract was split into two, and to one sample, buffer containing malonate acid was added, and to the other sample, an equal volume of buffer was added. Regions from the resulting 1H NMR spectra for the original and spiked samples are overlaid in blue and red, respectively. (PDF 1.03 mb) [file 40170_2016_155_MOESM3_ESM.pdf]

Figure S3B: 2D  $^1\text{H}$ - $^{13}\text{C}$ -HSQC spectra of a malonate spiked sample

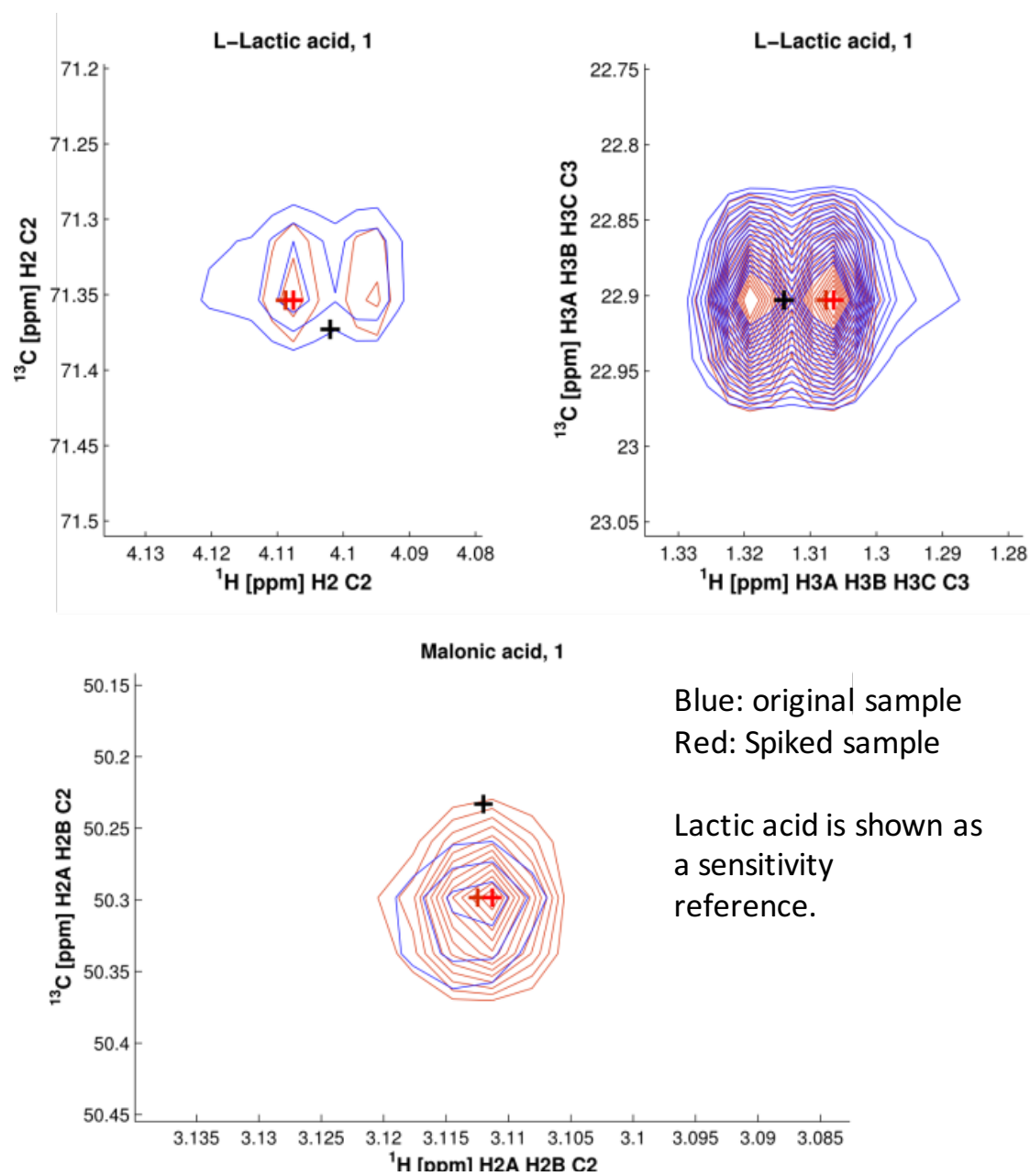

Supplement: Additional file 4: Figure S3B. — Malonate spiked sample. An unlabelled cell extract was split into two, and to one sample, buffer containing malonic acid was added and the pH adjusted to 7, and to the other sample, an equal volume of buffer was added. The regions of the resulting HSQC spectra containing the malonate and lactate resonances for the original and spiked samples are overlaid in blue and red, respectively. Lactate is shown as a sensitivity reference. (PDF 1.03 mb) [file 40170_2016_155_MOESM4_ESM.pdf]
