# Supplementary material for: Malonate as a ROS product is associated with pyruvate carboxylase activity in acute myeloid leukaemia cells
Source: Cancer Metab. 2016 Aug 4;4:15. doi: 10.1186/s40170-016-0155-7 (PMC4972992; doi:10.1186/s40170-016-0155-7)

Figure S4: Simulation of spectral multiplets arising from 1H-13C-HSQC spectra for aspartate and glutamate.

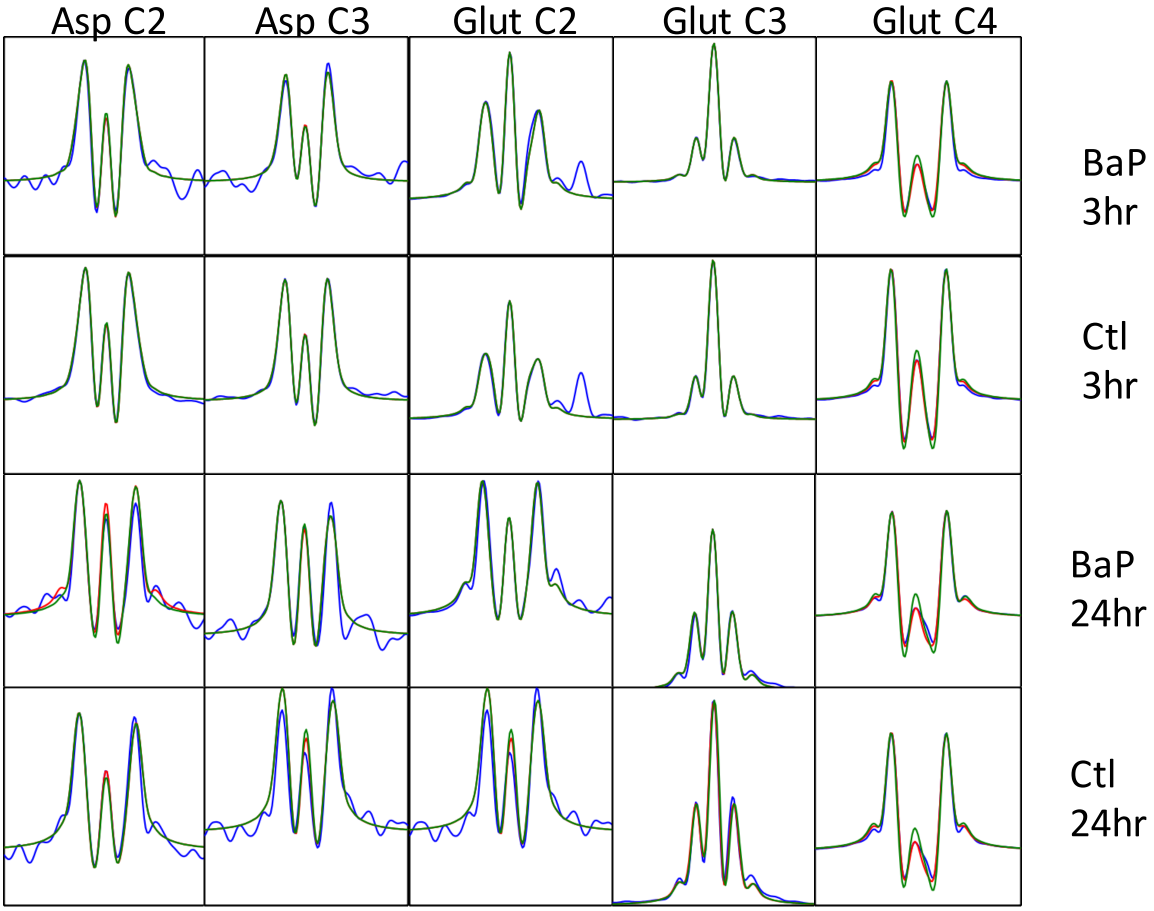

Supplement: Additional file 5: Figure S4. — Simulation of spectral multiplets arising from 1H-13C-HSQC spectra for aspartate and glutamate. (PDF 383 kb) [file 40170_2016_155_MOESM5_ESM.pdf]
